# Biophysical and Pharmacological Characterization of Nav1.9 Voltage Dependent Sodium Channels Stably Expressed in HEK-293 Cells

**DOI:** 10.1371/journal.pone.0161450

**Published:** 2016-08-24

**Authors:** Zhixin Lin, Sonia Santos, Karen Padilla, David Printzenhoff, Neil A. Castle

**Affiliations:** Neuroscience and Pain Research Unit, Pfizer Inc., Durham, North Carolina, United States of America; Dalhousie University, CANADA

## Abstract

The voltage dependent sodium channel Nav1.9, is expressed preferentially in peripheral sensory neurons and has been linked to human genetic pain disorders, which makes it target of interest for the development of new pain therapeutics. However, characterization of Nav1.9 pharmacology has been limited due in part to the historical difficulty of functionally expressing recombinant channels. Here we report the successful generation and characterization of human, mouse and rat Nav1.9 stably expressed in human HEK-293 cells. These cells exhibit slowly activating and inactivating inward sodium channel currents that have characteristics of native Nav1.9. Optimal functional expression was achieved by coexpression of Nav1.9 with β1/β2 subunits. While recombinantly expressed Nav1.9 was found to be sensitive to sodium channel inhibitors TC-N 1752 and tetracaine, potency was up to 100-fold less than reported for other Nav channel subtypes despite evidence to support an interaction with the canonical local anesthetic (LA) binding region on Domain 4 S6. Nav1.9 Domain 2 S6 pore domain contains a unique lysine residue (K799) which is predicted to be spatially near the local anesthetic interaction site. Mutation of this residue to the consensus asparagine (K799N) resulted in an increase in potency for tetracaine, but a decrease for TC-N 1752, suggesting that this residue can influence interaction of inhibitors with the Nav1.9 pore. In summary, we have shown that stable functional expression of Nav1.9 in the widely used HEK-293 cells is possible, which opens up opportunities to better understand channel properties and may potentially aid identification of novel Nav1.9 based pharmacotherapies.

## Introduction

Sodium flux through voltage-gated sodium (Nav) channels is a major contributor to action potential electrogenesis and propagation in excitatory cells [1–3]. Mammalian Nav channel family comprises nine isoforms (Nav1.1–1.9) that pair a pore-forming α-subunit with one non-covalently (β1 or β3) and one covalently (β2 or β4) auxiliary or beta subunit [2, 4, 5], which function to modulate channel cell surface expression and gating properties [6]. Several Nav channel subtypes like Nav1.7, Nav1.8 and Nav1.9 exhibit preferential expression in peripheral sensory neurons and have been reported to be important for conveying nociceptive sensory information from peripheral afferents to the central nervous system [7–12]. Because of their restricted expression profile and their proposed role in pain signaling, Nav1.7, Nav1.8 and Nav1.9 have received significant interest as potentially attractive targets for the development of novel pain therapeutics. While significant progress in the development of modulators of Nav1.7 and Nav1.8 has been reported [13] our understanding of the biology and pharmacology of Nav1.9 has developed more slowly, mainly due to an inability to functionally express recombinant forms of the channel in heterologous systems. Much of what we currently know about Nav1.9 has come from molecular and biophysical studies of endogenous Nav1.9 currents in sensory neurons, as well as characterization of transgenic mice lacking functional Nav1.9. These studies have shown that Nav1.9 has unique biophysical properties most notably activation at membrane potentials significantly more hyperpolarized than those required other neuronal Nav channels, and a much slower inactivation process results in persistent inward currents near the threshold membrane potential for action potential firing and possibly plays a role in regulating resting potential and amplifying depolarizing responses to subthreshold stimuli [9, 12, 14].

Recent advances in the identification of human genetic mutation of Nav1.9 associated with increased sensitivity to, or complete insensitivity to pain have amplified interest in this channel as a target for potential new pain medicine development. However, the historical difficulty of functional expression of recombinant Nav1.9 in heterologous systems has hindered the systematic investigation of channel properties and has made the identification of pharmacological modulators challenging. In the past couple of years there have been reports of successful stable expression of human Nav1.9 in transformed sensory neuron neuroblastoma hybridoma which have enabled some of the biophysical properties of recombinant Nav1.9 to be compared with endogenous Nav1.9 currents [15]. However, there remains a paucity of scientific literature describing Nav1.9 pharmacology. In the current study we describe the first successful, to our knowledge, stable expression of functional human and rodent isoforms of Nav1.9 in HEK-293 cells. This expression system is suitable for studying biophysical properties of Nav1.9 and for characterizing pharmacology and identifying new modulators. We have also been able to generate Nav1.9 mutants to enable better understanding of compound interaction with the channel.

## Results

### Stable expression of human Nav1.9 in HEK-293 cells

**Fig 1:**
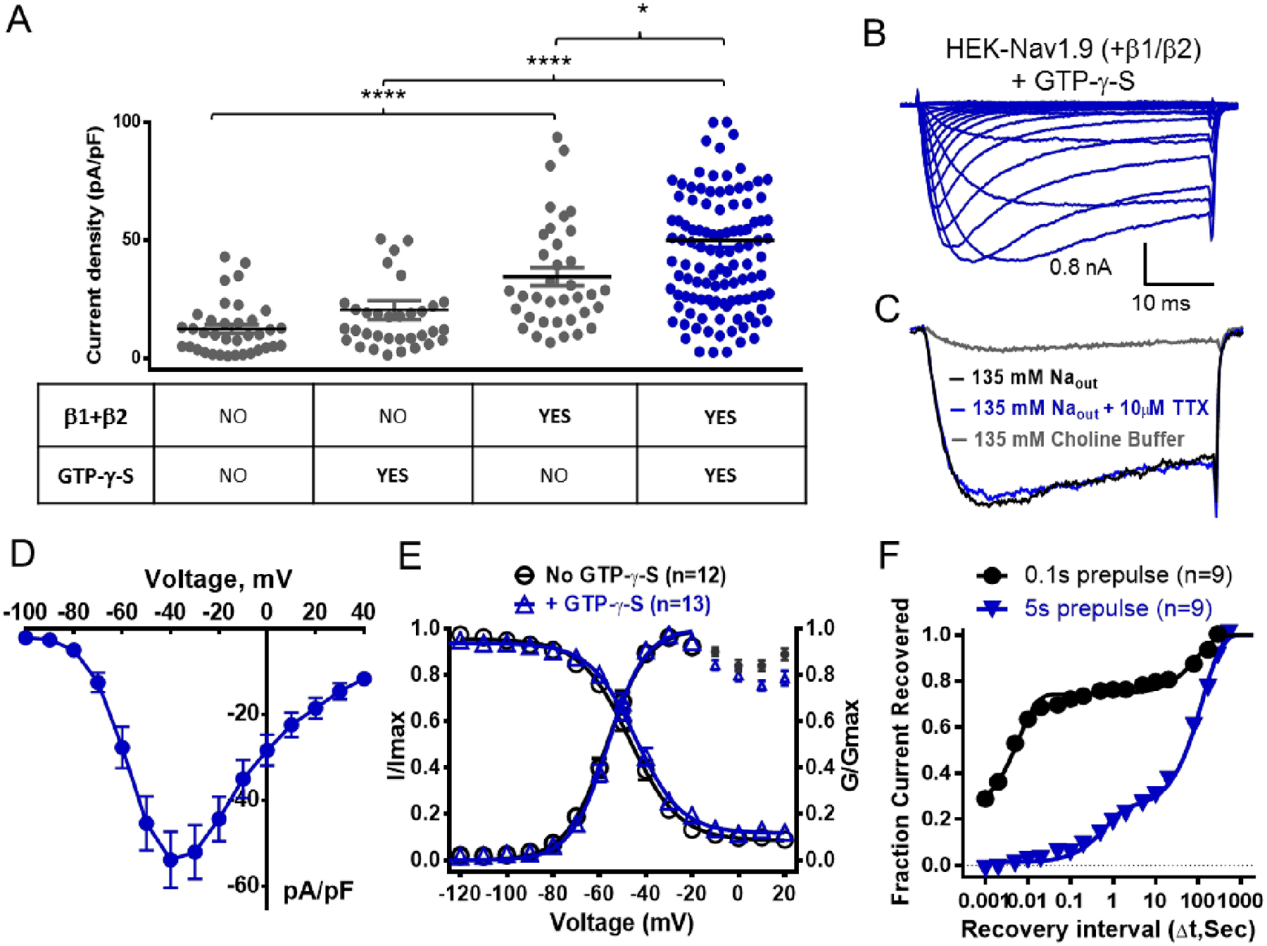
Expression and biophysical properties of human Nav1.9 stably expressed in HEK-293 cells. (A) Comparison of peak current density (pA/pF) of inward currents elicited by 40 ms voltage steps from -140 mV to -40 mV in HEK-293 cells stably expressing Nav1.9 alone or coexpressed with β1 and β2 subunits in presence and absence of 500 μM GTP-γ-S in intracellular solution. (B) Representative family current traces recorded in HEK-293 cells expressing Nav1.9 + β1/β2 recorded with 500 μM GTP-γ-S in pipette following a series of incremental voltage steps from –140 mV to +40 mV. (C) Inward current traces elicited in Nav1.9 + β1/β2 by 40 ms voltage steps from -140 mV to -40 mV in extracellular buffer containing 135 mM Na^+^ in absence or presence 10 μM tetrodotoxin (TTX) or in an extracellular buffer where all the sodium was replaced by choline. (D) Current density voltage relationship recorded in HEK-293 cells stably expressing Nav1.9 + β1/β2 + 500 μM GTP-γ-S (data are mean ± SEM from n = 13 observations). (E) Voltage dependence of activation and inactivation of Nav1.9 + β1/β2 recorded in the presence and absence of 500 μM GTP-γ-S following incremental depolarizing 100 ms conditioning voltage steps from -140 mV followed by a 40 ms test pulse to -40 mV. Data was fitted to a Boltzmann equation with parameters shown in Table 2. (F) Time course of recovery inactivation induced by either a 100 ms (circle) or 5 s (triangle) depolarizing voltage step to 0 mV. Recovery was assessed by applying a 40 ms test pulse to -40 mV after variable periods at -140 mV. Data was fit with a two phase exponential equation with fitted fast and slow time constants shown in Tables 3 and 4.

The initial goal of the current study was to identify conditions in which human Nav1.9 could be functionally expressed in the widely used HEK-293 heterologous expression system. While a number of attempts to express the Nav1.9 alpha subunit alone did result in cell lines that expressed significant Nav1.9 mRNA levels, the proportion of cells exhibiting functional inward currents elicited by 100 ms voltage command steps from -140 mV to -40 mV with characteristics of Nav1.9 ranged from zero to at best 5%. Furthermore, when present, current amplitudes were generally less than 100 pA. Improvement in inward current density was achieved by preincubating cells at 28°C overnight prior to experimental evaluation (12 ± 1 pA/pF), although only 33% of the cells expressed currents >200 pA (See Fig 1A, Table 1). A further boost in Nav1.9 current density (20 ± 4 pA/pF) and percentage of cells expressing current (59% >200 pA) was achieved by including 500 μM GTP-γ-S in the intracellular solution (Fig 1A, Table 1) which is consistent with findings from previous studies with native and recombinant Nav1.9 [15–18]. Despite these improvements, stable cell lines expressing Nav1.9 alpha subunit alone were not considered robust or consistent enough for our experimental needs. Given that in their native environment, Nav channels typically comprise of both the alpha pore forming subunits and two auxiliary beta subunits, we investigated the effect of coexpressing human Nav1.9 alpha subunit with human β1 and β2 subunits. To do this, we took the approach of initially generating a cell line stably expressing stoichiometric equivalent amounts β1 and β2 subunits. This was achieved by creating a concatemer cDNA using a 18 amino acid T2A-linker [19] to link β1 and β2 subunit sequences, so that the β1-T2A-β2 are generated from the same mRNA, to produce two independent proteins. Clonal HEK-293 cell lines with β1 and β2 mRNA levels equivalent to or greater than the housekeeping gene GAPDH were identified by qPCR. One of the β1/β2 expressing stable cell lines was then infected with human Nav1.9 cDNA and functional Nav1.9 expressing clonal cell lines were again identified by the presence of slowly inactivating inward sodium currents elicited by a voltage steps from -140 mV to -40 mV using the PatchXpress automated patch clamp platform. Current density of Nav1.9 currents in β1/β2 expressing cells was 35 ± 4 pA/pF (Fig 1A) which is 2–3 fold greater than observed with the alpha subunit alone. Furthermore, 85% of the cells expressed Nav1.9 currents >200 pA. Maximal current density was achieved by incubating HEK-Nav1.9 β1/β2 cells overnight at 28°C in the presence of GTP-γ-S in the recording pipette (50 ± 3 pA/pF, 88% cells >200pA). Fig 1B shows a family of inward current traces elicited by incrementally more depolarized voltage steps from a holding potential of -140 mV recorded in HEK-Nav1.9 β1/β2 cells under the above described optimized conditions. Inward currents activated and inactivated with relatively slow kinetics (compared to other Nav channel subtypes). Peak inward current amplitude/density was observed at -40 mV, and was insensitive to inhibition by 10 μM tetrodotoxin but was almost completely abolished following the replacement of extracellular sodium with cell membrane impermeant choline (Fig 1C). Fig 1D illustrates the current density voltage relationship for Nav1.9 recorded under optimized conditions. All of the observed characteristics are consistent with the known properties of native Nav1.9. Fig 1C shows the voltage-dependence of Nav1.9 channel gating in the presence and absence of GTP-γ-S. Although the presence of GTP-γ-S in recording pipette was associated with an increase in Nav1.9 current density, it appears to have no obvious effects on either voltage dependence of activation (midpoint; -57 ± 0.4 mV and -56 ± 0.4 mV with and without GTP-γ-S respectively) or inactivation (midpoint -47 ± 0.9 mV and -46 ± 1 mV in presence and absence of GTP-γ-S respectively) (Table 2). The time course for Nav1.9 recovery from inactivation is shown in Fig 1F. When Nav1.9 inactivation was evoked by 100 ms voltage step to 0 mV current amplitude recovered with a biexponential time course with a rapid phase (τ = 6 ms) and a smaller slower phase (τ = 98 s) (see Table 3 for summary of parameters). In contrast, when inactivation was elicited using a 5 second conditioning pulse to 0 mV, time course for recovery of current was much slower, with the main component recovering with τ = 130 s and full recovery taking ~10 min. (see Table 4 for summary of biophysical parameters).

**Table 1:** Current Density of HEK-293 hNav1.9 Stable Cell Lines.

β1/2 Subunits Expressed	Overnight Cell Culture Temp	500 μM GTP-γ-S Present in Recording Pipette	Nav1.9 Current Density (pA/pF)	% Cells with Current Amplitude >200 pA	n
No	28°C	No	12 ± 1	33	36
No	28°C	Yes	20 ± 4	59	34
Yes	28°C	No	35 ± 4	84	38
Yes	28°C	Yes	50 ± 3	88	120
Yes	37°C	No	26 ± 4	53	32
Yes	37°C	Yes	30 ± 3	77	35

**Table 2:** Voltage Dependence of Activation and Inactivation.

	Activation		Inactivation			
	V_0.5_ (mV)	k	V_0.5_ (mV)	k	Prepulse Duration (ms)	n
**hNav1.9 (no GTP-γ-S)**	-57.2 ± 0.7	7.7 ± 0.6	-46.9 ± 0.9	10.6 ± 0.8	100	12
**hNav1.9**	-56.2 ± 0.5	7.3 ± 0.5	-46.3 ± 1.0	12.7 ± 0.8	100	13
**mouse Nav1.9**	-62.6 ± 0.6	6.2 ± 0.5	-55.4 ± 0.6	9.6 ± 0.5	100	12
**rat Nav1.9**	-66.3 ± 0.4	7.6 ± 0.4	-58.8 ± 0.6	12.7 ± 0.5	100	21
**hNav1.9**	-50.9 ± 0.7	7.2 ± 0.6	-49.5 ± 0.6	9.5 ± 0.5	500	9
**hNav1.9 F1592/Y1599A**	-55.5 ± 0.5	7.6 ± 0.6	-55.8 ± 0.7	9.0 ± 0.6	500	10
**hNav1.9 K799N**	-47.5 ± 0.9	7.1 ± 0.7	-53.6 ± 0.7	10.1 ± 0.7	500	14
**hNav1.7**	-25.5 ± 0.4	5.7 ± 0.3	-65.0 ± 0.3	5.1 ± 0.3	500	10
**hNav1.7 N945K**	-25.7 ± 0.4	5.0 ± 0.3	-63.8 ± 0.3	4.8 ± 0.3	500	9

**Table 3:** Time Constants for Recovery from Inactivation Induced by 100 ms Prepulse to 0 mV.

**hNav1.9**	**τ**_**fast**_**(s)**	**0.006**	**τ**_**slow**_**(s)**	**98**
	95% CL*	0.005 to 0.008	95% CL	75.3 to 139.1
**hNav1.9 K799N**	**τ**_**fast**_**(s)**	**0.006**	**τ**_**slow**_**(s)**	**90**
	95% CL	0.005 to 0.010	95% CL	58.9 to 191.6

*95% confidence limits, n = 9 observations

**Table 4:** Time Constants for Recovery from Inactivation Induced by 5 s Prepulse to 0 mV.

**hNav1.9**	**τ**_fast_**(s)**	**0.6**	**τ**_**slow**_**(s)**	**130**
	95% CL*	0.5 to 0.9	95% CL	121.7 to 139.1
**hNav1.9 K799N**	**τ**_**fast**_**(s)**	**1.9**	**τ**_**slow**_**(s)**	**97**
	95% CL	1.2 to 4.5	95% CL	78.4 to 128.3

*95% confidence limits, n = 9 observations

### Pharmacological properties of HEK-hNav1.9

**Fig 2:**
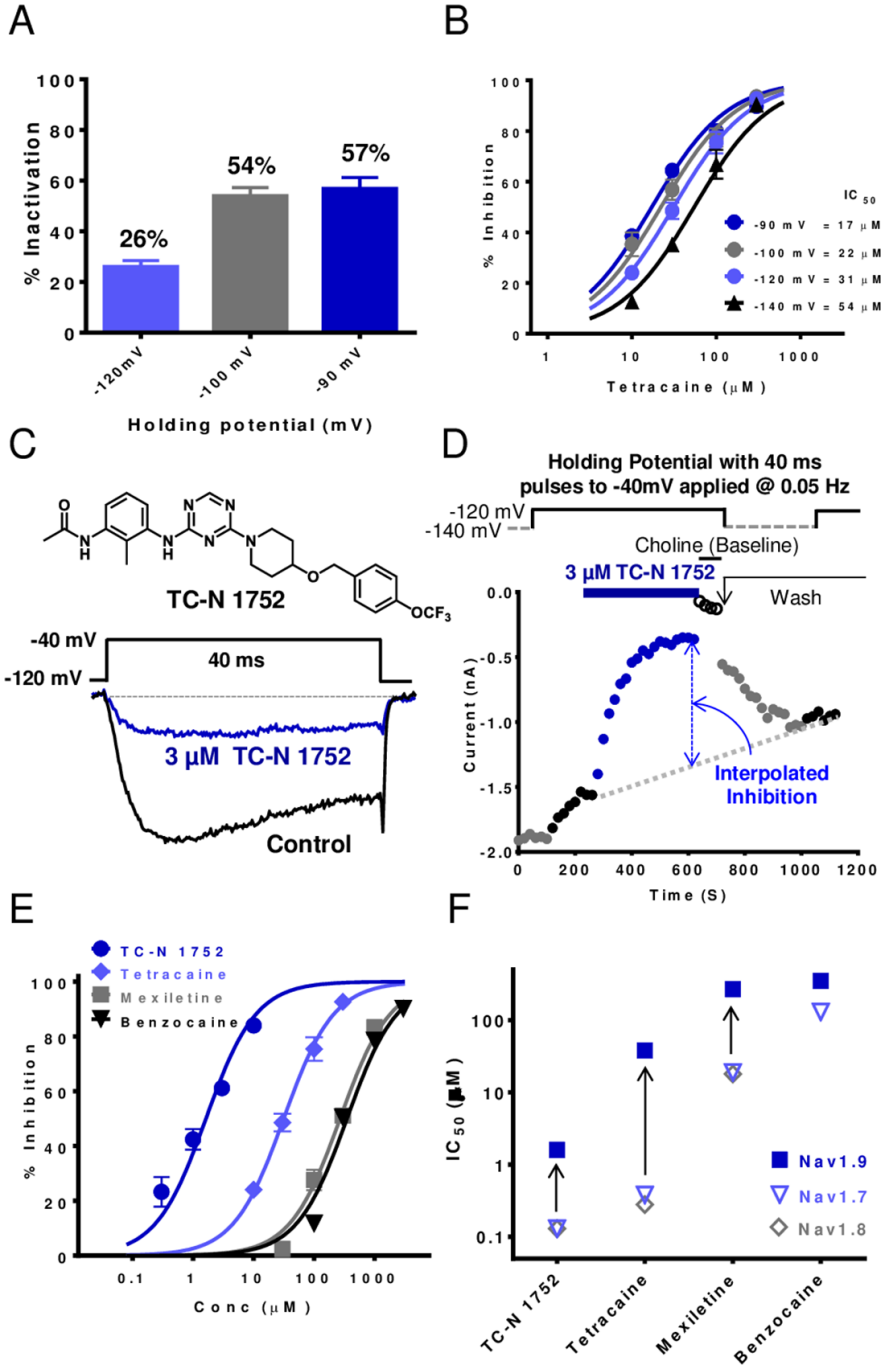
Pharmacological modulation of human Nav1.9. (A) Magnitude of Nav1.9 inactivation at different holding potentials. Percent inactivation determined from decline in test pulse current amplitude applied every 20s from test holding potential to -40 mV (values are mean ± SEM for 11–12 observations). (B) Comparison of concentration dependence of Nav1.9 inhibition by tetracaine at different holding potentials. (C) Representative current traces recorded in HEK-hNav1.9 cell line in the presence and absence of 3 μM TC-N 1752 (structure shown) during a 40 ms voltage step from -120 mV to -40 mV. (D) Diary plot of Nav1.9 current amplitude recorded using 40 ms voltage steps to -40 mV applied at 0.05 Hz, initially from a holding potential of -140 mV, then at -120 mV to induce ~25% inactivation. After current amplitude was stable at -120 mV, test agent was applied. Washout of test agent at holding potential of -140 mV until current amplitude became stable and then holding potential reset to -120 mV to allow inhibition to be determined by dividing current amplitude in presence of compound by interpolated current amplitude (see dotted line) between prior to compound addition and after washout. (E) Concentration response relationships for inhibition of Nav1.9 currents by known Nav channel inhibitors TC-N 1752, tetracaine, mexiletine and benzocaine. IC_50_s for these agents are plotted in (F) (values are summarized in Table 5) and are compared to IC_50_s for inhibition of Nav1.7 and Nav1.8 (values are summarized in Table 6).

Pharmacological modulators of sodium channels often interact in a gating state dependent manner, with many inhibitors preferentially interacting with the inactivated conformation [20, 21]. Examination of Nav1.9 inhibition, particularly with respect to the presence or absence of inactivation is complicated by the slow recovery from inactivation illustrated in Fig 1E. When the membrane potential was clamped to voltages more depolarized than -140 mV inactivation became evident (see Fig 2A) with approximately a 50% reduction in available current at -90 mV, suggesting that the voltage dependence of slow inactivation is significantly more hyperpolarized than inactivation elicited by 100 ms conditioning pulses shown in Fig 1E. Fig 2B shows concentration dependence of inhibition of Nav1.9 currents by the local anesthetic agent tetracaine at holding potentials extending from -140 mV to -90 mV. Tetracaine’s IC_50_ ranged from 54 μM at -140 mV to 17 μM at -90 mV, reflecting an apparent 3 fold increase in potency at the most depolarized membrane potential. This increase in potency is considerably smaller than the shift reported with local anesthetic block of other Nav channels [22, 23]. Furthermore, we cannot exclude the possibility that a component of the reduced current in the presence of tetracaine at depolarized potentials results from an accumulation of inactivation (“rundown”) due to slow recovery from inactivation noted above. To minimize the impact of the observed current “rundown”, while maintaining some channel inactivation, pharmacological studies were performed using a protocol in which the holding potential was initially set to -140 mV to assess magnitude of available current, then switched to -120 mV to introduce ~20–25% inactivation. Current amplitude before and after application of test compound was determined by a 40 ms voltage step to -40 mV applied every 20 s. Representative current traces recorded in the presence and absence of 3 μM TC-N 1752 are shown in Fig 2C. Inhibition of Nav1.9 by the sodium channel inhibitor TC-N 1752 using this protocol is shown in Fig 2D along with the time course for onset and washout effect. Magnitude of inhibition was calculated by dividing current amplitude in presence of inhibitor by the average of current amplitude before compound addition and after compound wash off (see Fig 2D). The concentration dependence of Nav1.9 inhibition by TC-N 1752, tetracaine, mexiletine and benzocaine determined using the above protocol is shown in Fig 2E. Fitted IC_50_s for these and other pharmacological agents are shown in Table 5. Fig 2F compares the IC_50_s for inhibition of Nav1.9 with IC_50_s for human Nav1.7 and Nav1.8 by TC-N 1752, tetracaine, mexiletine and benzocaine. In contrast to Nav1.7 and Nav1.8, which exhibited similar sensitivities to these inhibitors, Nav1.9 was considerably less sensitive to three of the agents, with tetracaine, mexiletine and TC-N 1752 being 100, 14 and 12 fold less potent respectively.

**Table 5:** Potencies for Inhibition of Nav1.9 and Nav1.9 F1592A/Y1599A.

	Human Nav1.9			Human Nav1.9 F1592A/Y1599A		
Compounds	IC_50_ (μM)	95% CI	n	IC_50_ (μM)	95% CI	n
**Amitriptyline**	29	25 to 34	6 to 11	105	84 to 131	6 to 7
**Benzocaine**	351	300 to 411	6 to 10	1757	1462 to 2112	6
**Bupivacaine**	186	144 to 240	6 to 7			
**Flecainide**	244	208 to 286	6 to 8			
**Lamotrigine**	199	168 to 235	6 to 10			
**Lidocaine**	356	309 to 411	5 to 11	5553	3302 to 9339	6 to 9
**Mexiletine**	270	231 to 315	6 to 9	837	697 to 1005	6
**TC-N 1752**	1.6	1.4 to 1.9	6 to 12	273	119 to 627	6
**Tetracaine**	32	20 to 36	9 to13	175	147 to 209	6 to 9
**TTX**	NA @ 10 μM		4			
**A-803467**	30% @ 10 μM		4			
**ICA-121431**	NA @ 10 μM		4			
**CdCl**_**2**_	NA @ 100 μM		3			
**Mibefradil**	NA @ 10 μM		3			
**Nimodipine**	33	12 to 90	3			

NA = not active

**Table 6:** Potencies for Inhibition of Nav1.7 and Nav1.8.

	Human Nav1.7			Human Nav1.8		
Compounds	IC_50_ (μM)	95% CI	n	IC_50_ (μM)	95% CI	n
**TC-N 1752**	0.2	0.1 to 0.2	6 to 9	0.1	0.1 to 0.2	12 to 18
**Tetracaine**	0.4	0.3 to 0.4	7	0.3	0.2 to 0.4	3 to 13
**Mexiletine**	19	16 to 23	4 to 15	18	12 to 26	3 to 4
**Benzocaine**	133	114 to 157	6 to 8			

### Canonical local anesthetic binding site contributes to the interaction of Nav1.9 channel inhibitors

**Fig 3:**
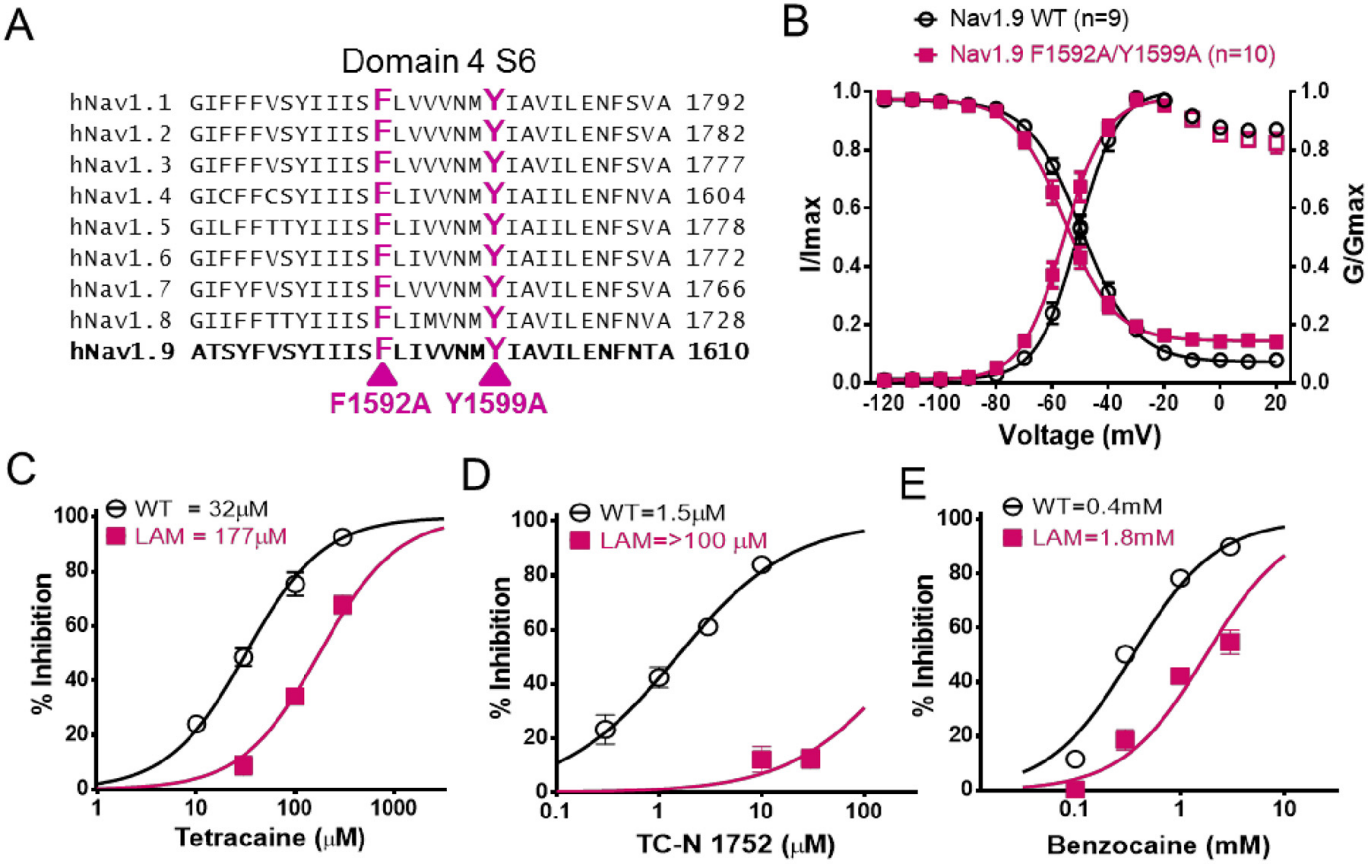
Interaction of sodium channel inhibitors with Nav1.9 canonical local anesthetic binding site. (A) Sequence alignment of D4 S6 segments for Nav1.1 to Nav1.9. The canonical local anesthetic binding site residues phenylalanine and tyrosine are labeled in magenta and were mutated to alanine (F1592A/Y1599A) to assess their contribution to Nav1.9 inhibition. (B) Comparison of the voltage dependence of activation (Nav1.9 WT: -51 ± 1 mV, n = 9; Nav1.9 F1592A/Y1599A: -56 ± 1 mV, n = 10; P>0.05) and inactivation following a 500 ms conditioning prepulse (WT: -50 ± 1 mV, n = 9; Nav1.9 F1592A/Y1599A: -56 ± 1 mV, n = 10; P<0.05). Comparison of concentration response relationships for inhibition of Nav1.9 WT (open symbols) and Nav1.9 F1592A/Y1599A (Closed symbols, LAM) by tetracaine (C), TC-N 1752 (D), and benzocaine (E). Fitted IC_50_s are summarized in Table 5.

The lower potency of traditional local anesthetic like agents for inhibition of Nav1.9 raised the question of whether these compounds interact with the canonical local anesthetic pore binding region on homologous domain 4 S6 of Nav1.9. Fig 3A shows the amino acid sequence alignment for this region for all Nav channel subtypes and illustrates that Nav1.9 amino acid residues are very similar to other subtypes, including the conserved canonical local anesthetic binding site residues Phenylalanine (F) and Tyrosine (Y) indicated. We generated a stable Nav1.9 cell line in which the F1592 and Y1599 residues were mutated to alanine (Nav1.9 F1592A/Y1599A, Fig 3A) which has been shown to reduce local anesthetic potency for inhibition of other Nav channel subtypes [21, 22, 24]. The biophysical properties of hNav1.9 F1592A/Y1599A compared to Nav1.9 are shown in Fig 3B. The mid-point potential for the voltage-dependence of activation was: -51 ± 1 mV, n = 9 and -56 ± 1 mV, n = 10; P>0.05 for Nav1.9 and Nav1.9 F1592A/Y1599A respectively, and for inactivation Nav1.9 LAM: -56 ± 1 mV n = 10; Nav1.9: -50 ± 1 mV, n = 9; P<0.05. The effect of the Nav1.9 F1592A/Y1599A mutation on tetracaine, benzocaine and TC-N 1752 mediated inhibition is shown in Fig 3C, 3D and 3E. For all compounds potency was reduced, with local anesthetic agents, tetracaine and benzocaine exhibiting 5.6 and 5 fold reductions in IC_50_s, respectively. The largest decrease in potency was observed for TC-N 1752 which exhibited a >180 fold decrease in potency. This latter finding provides the first evidence for a potential local anesthetic binding site interaction for this structural class of sodium channel inhibitor. The IC_50_s and shifts in potency for inhibition of Nav1.9 F1592A/Y1599A for the above compounds and several additional inhibitors are summarized in Table 5.

### Lysine 799 (Domain 2 S6) unique to Nav1.9 can influence modulation by inhibitors

**Fig 4:**
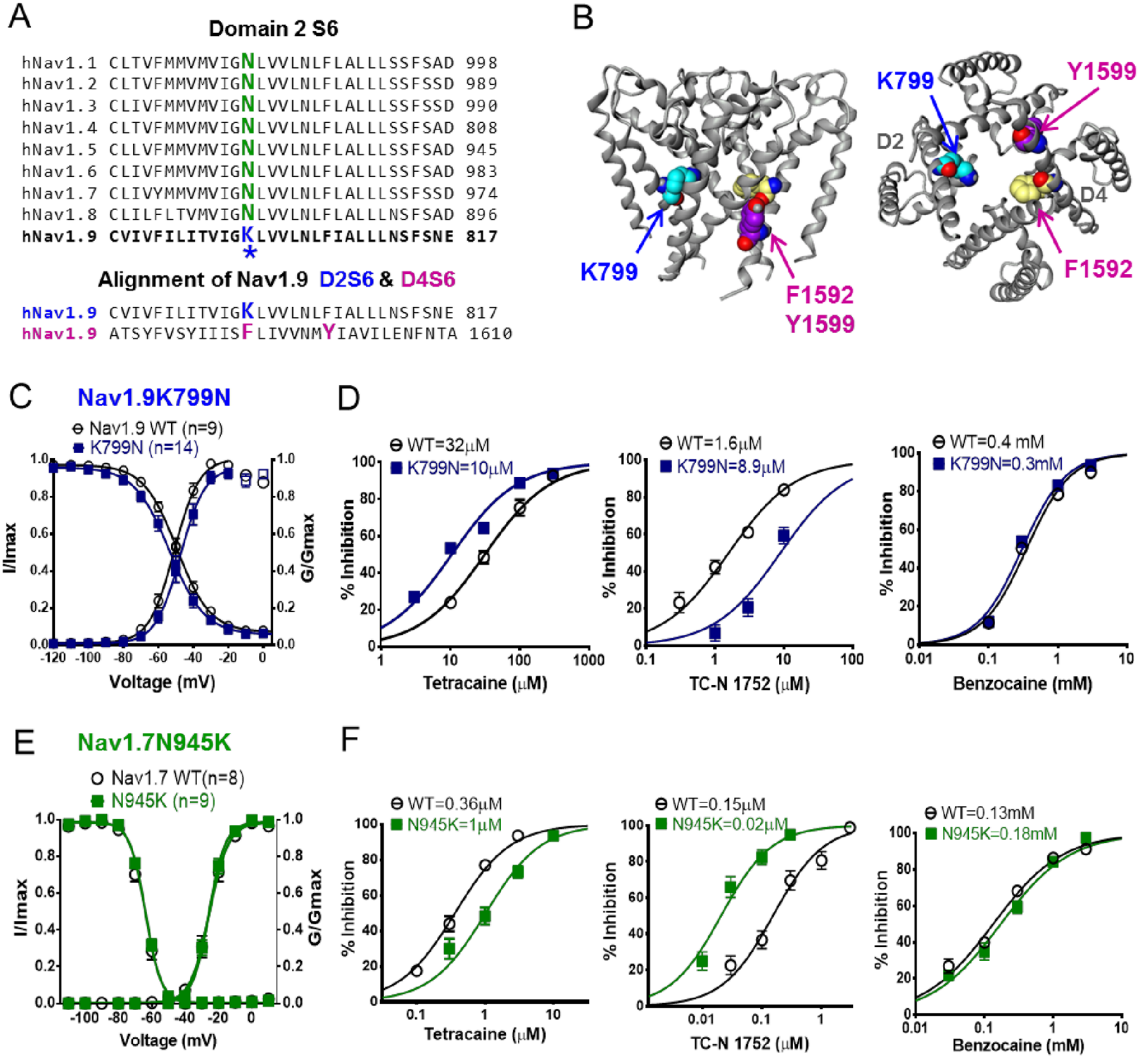
Characterization of K799 residue unique to Nav1.9 in Domain 2 S6. (A) Upper panel; sequence alignment of D2 S6 for Nav1.1 to Nav1.9. Lysine residue K799 is labeled in blue whereas the consensus asparagine found at the equivalent position in other Nav channel subtypes is shown in green. Lower panel; sequence alignment of Nav1.9 D2 S6 with D4 S6 segments showing position of K799 residue relative to F1592 and Y1599 residues that form part of the canonical local anesthetic binding site. (B) Homology model of human Nav1.9 based on X-ray crystal structure of NavM [26] illustrating the position of lysine K799 in relation to F1592 and Y1599. (C) Comparison of the voltage dependence of activation (Nav1.9 WT: -51 ± 1 mV, n = 9; K799N: -48 ± 1 mV, n = 14; P>0.05) and inactivation induced by 500ms conditioning prepulse (Nav1.9 WT: -50 ± 1 mV, n = 9; K799N: -54 ± 1 mV, n = 14; P>0.05). (D) Comparison of concentration dependence of inhibition of Nav1.9 WT and Nav1.9 K799N channels by tetracaine, TC-N 1752 and benzocaine. Potency was determined using protocol described in Fig 2. (E) Comparison of the voltage dependence of activation and inactivation of Nav1.7 WT and Nav1.7 N945K mutant channels (activation; WT: -26 ± 0.4 mV, n = 10; N945K: -26 ± 0.4 mV, n = 9; P>0.05; and inactivation, WT: -65 ± 0.3 mV, n = 10; N945K: -64 ± 0.3 mV, n = 9; P>0.05) using same conditioning prepulse protocol as in (C) except utilizing a 20 ms test pulse to 0 mV. (F) Comparison of concentration dependence of inhibition of Nav1.7 WT and Nav1.7 N945K channels by tetracaine, TC-N 1752 and benzocaine.

The amino acid sequence of human Nav1.9 differs significantly from other Nav channels subtypes but there are regions where the sequence exhibits high conservation, most notably helices that form the channel pore. Within these conserved pore sequences, Nav1.9 has a number of unique residues. Of particular interest is the positively charged lysine at position 799 (K799) in the S6 transmembrane segment of homologous domain 2. The equivalent residue in all other Nav channel subtype is asparagine (see Fig 4A). K799 is next to a glycine proposed to form part of the gating “hinge” [25], and a homology model of Nav1.9 based on the crystal structure of NavM bacterial sodium channel [26] places the residue spatially directly opposite across the pore from the F1592 residue on Domain 4 S6 which forms part of the canonical local anesthetic binding site (see sequences alignment in Fig 4A and Nav1.9 homology model based on X-ray crystal structure of NavM [26] in Fig 4B). Given its charge and location within the pore, we investigated if K799 influences inhibitor interaction with Nav1.9, in particular compounds that may interact with the local anesthetic binding site. To do this we constructed the mutant HEK-hNav1.9K799N stable cell line, in which the lysine was replaced with the asparagine found in all other Nav subtypes at this equivalent location (see Fig 4A). The Nav1.9-K799N cell line exhibited similar biophysical properties to wild type Nav1.9 with respect to the voltage-dependence of activation (WT: -51 ± 1 mV, n = 9; K799N: -48 ± 1 mV, n = 14; P>0.05) and steady state inactivation (WT: -50 ± 1 mV, n = 9; K799N: -54 ± 1 mV, n = 14; P>0.05) (Fig 4C). The time course of recovery from inactivation was also similar to wild type Nav1.9 (see S1 Fig). To evaluate the impact of the K799N mutation on Nav1.9 pharmacology we examined potency of TC-N 1752 and the two local anesthetic agents, tetracaine and benzocaine since we have shown their effect is modulated by the local anesthetic binding site mutation, suggesting a pore interaction. Furthermore, by comparing the neutral local anesthetic benzocaine with tetracaine, which is predominantly positively charged at physiological pH, we had an opportunity to evaluate the potential influence of the positive charge of K799. Fig 4D compares concentration response curves for all three agents for inhibition of Nav1.9 K799N vs Nav1.9. Benzocaine potency was unaffected by K799N mutation whereas, tetracaine potency was increased 3 fold from 31.7 to 10.3 μM. In contrast, potency of TC-N 1752 was decreased 5.6 fold from 1.6 to 8.9 μM. To increase our confidence that the disparate and relatively small shifts in potency observed were meaningful, we explored the expectation that mutating the consensus asparagine residue to lysine in other Nav channel subtypes might have opposite effects to those observed in Nav1.9. To do this, we generated a stable mutant Nav1.7 cell line (Nav1.7 N945K) carrying the equivalent Nav1.9 residue. The voltage-dependence of activation and inactivation of Nav1.7 N945K were similar to that of wild type Nav1.7 channels (Fig 4E, Table 2). As shown in Fig 4F Nav1.7 N945K exhibited no shift in sensitivity to benzocaine compared to the wild type Nav1.7 channel, similar to that observed with wild type Nav1.9 and Nav1.9 K799N. However, Nav1.7 N945K exhibited a 3 fold decrease in sensitivity to tetracaine and an 8 fold increase in potency for inhibition by TC-N 1752 compared to wild type Nav1.7, which is the opposite of what was observed with the reverse mutation at the equivalent location on Nav1.9.

### Stable expression and characterization of rodent Nav1.9

**Fig 5:**
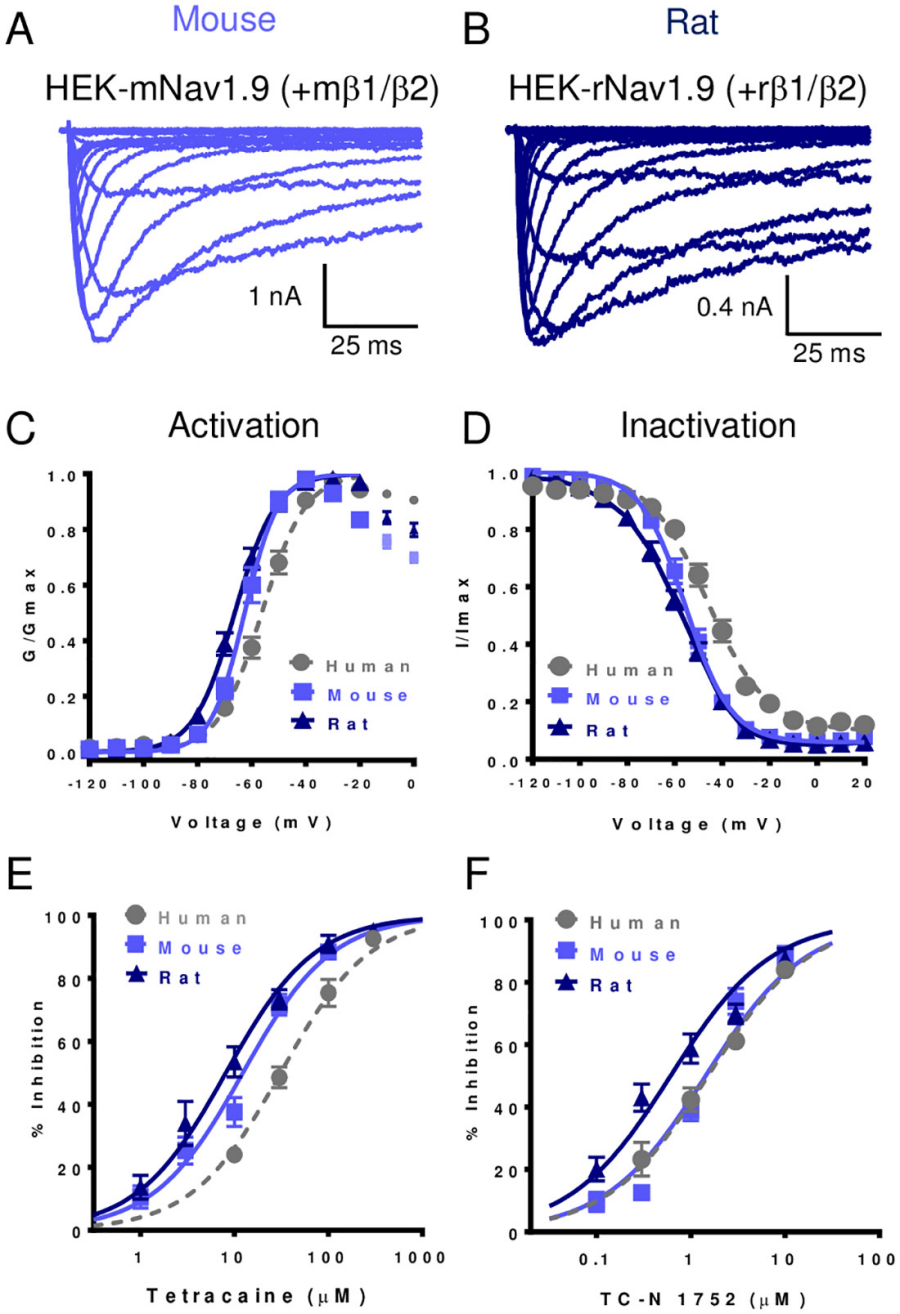
Expression and biophysical properties of rodent Nav1.9 stably expressed in HEK-293 cells. Representative family of current traces recorded in HEK-293 cells stably expressing, (A) mouse Nav1.9 plus mouse β1/β2 or (B) rat Nav1.9 plus rat β1/β2. (C) Comparison of voltage dependence of activation for human, mouse and rat Nav1.9. (D) Comparison of steady-state inactivation following 100 ms conditioning prepulse for human, mouse and rat Nav1.9. Parameters for both activation and inactivation derived from fits of the Boltzmann equation are summarized in Table 2. (E) Comparison of concentration dependence of inhibition of human, mouse and rat Nav1.9 by tetracaine. (F) Comparison of concentration dependence of inhibition of human, mouse and rat Nav1.9 by TC-N 1752. Data points for (E) and (F) are means ± SEM for n = 4 to 13 observations. IC_50_s derived from fitting data are summarized in Table 7.

Rats and mice are used widely in efficacy and toxicity assessment during drug development. Given that rat and mouse Nav1.9 share only 76% identity to human Nav1.9 and there are increasing reports of differential Nav channel pharmacology for species orthologs [27, 28] we constructed stable cell lines and characterized rat and mouse species orthologs of the channel. Employing the same strategy used for human Nav1.9, rat and mouse Nav1.9 stable cell lines were generated by coexpressing the alpha subunit relevant species versions of β1 and β2 subunits. Fig 5A and 5B show that mouse and rat HEK-Nav1.9 cell lines generated in this manner exhibited robust functional expression. The voltage dependence of activation and inactivation of rat and mouse Nav1.9 were found to be similar to each other but slightly more hyperpolarized than human Nav1.9 (Fig 5C, 5D and Table 2). Fig 5E and 5F compare the concentration dependence of inhibition of human, mouse and rat Nav1.9 by tetracaine and TC-N 1752. For both compounds, potencies for inhibition of rodent Nav1.9 were 3–4 fold greater than for human Nav1.9 (Table 7).

**Table 7:** Potencies for Inhibition of Rat and Mouse Nav1.9.

	Rat Nav1.9			Mouse Nav1.9		
Compounds	IC_50_ (μM)	95% CI	n	IC_50_ (μM)	95% CI	n
**TC-N 1752**	0.5	0.4 to 0.7	5 to 8	1.4	1.1 to 1.9	4 to 8
**Tetracaine**	7.6	5.5 to 10	4 to 15	12	9.8 to 15	4 to 6

## Discussion/Conclusions

In the present study we report the first successful stable functional expression and characterization of human Nav1.9 in HEK-293 cells that demonstrate biophysical similarities to that reported from native Nav1.9 currents observed in sensory neurons [18, 29]. In addition, we show pharmacological divergence in some pan-Nav channel inhibitor chemotypes compared to the other peripherally expressed Nav1.7 and Nav1.8 channels. Despite its original cloning over 18 years ago, stable expression and characterization of recombinant Nav1.9 has proved challenging and in many cases unsuccessful. While there have been historical reports of stable Nav1.9 currents following coexpression with TrkB receptor [30] the characteristics of these currents were quite distinct from native Nav1.9 currents, which raised uncertainty as to their true association with Nav1.9. There have been studies showing an increase in Nav1.9 protein following coexpression with proteins like contactin [31], although there were no reports of a similar up-regulation of function Nav1.9-like currents. Addition of Glial-Derived Neurotrophic Factor (GDNF) to culture media increased expression of functional Nav1.9-like currents in axotomized dorsal root ganglion neurons [32]. More recently, Vanoye et al. reported the stable expression of Nav1.9 in the rat sensory neuron/mouse neuroblastoma hybridoma cell line, ND7/23 [15]. Studies using this cell system showed that increase functional expression of human Nav1.9 could be achieved by growing cells at 28°C rather than 37°C prior to experimental testing, presumably via an increase in trafficking similar to that reported for hERG and CFTR [33, 34]. They also reported a boost in functional expression by including GTP-γ-S in the recording pipette. This finding is consistent with the amplification of sensory neuron Nav1.9 current expression following treatment with G-protein receptor ligands found in endogenous inflammatory conditions [16, 17, 35]. In the current study reported here we also found that incubating at 28°C and including GTP-γ-S in the intracellular recording solution enhanced expression of functional Nav1.9 in HEK-293 cells, but not sufficiently to make the cell line experimentally useful with the alpha subunit alone. The single largest increase in functional expression of Nav1.9 came when we coexpressed the alpha subunit with both β1/β2 subunits. This was true for both human Nav1.9 and rodent orthologs. Indeed, for HEK-Nav1.9 β1/β2, robust currents can be recorded in the absence of preincubating at 28°C or administering GTP-γ-S (Table 1).

During our evaluation of the biophysical properties of human Nav1.9 we found many similarities to that reported for endogenous Nav1.9 in sensory neurons. HEK-Nav1.9 currents activate and inactivate slowly and at membrane potentials more hyperpolarized than other Nav channel subtypes. While the presence of GTP-γ-S increases current density it has no obvious effect on voltage dependence of gating. One intriguing observation that came out of the biophysical analysis is the slow time course for recovery from inactivation. For short periods of inactivation (~100–500 ms) the midpoint potential is around ~ -50 mV and recovery occurs for the most part within 1 s. However, when inactivation is produced by long depolarizing membrane potentials (>5 s), the midpoint of inactivation is closer to ~ -90 mV and recovery takes up to 10 minutes for currents to regain maximal amplitude. This extraordinary long recovery from inactivation presented some challenges for experimental studies especially pharmacology, since repetitive pulses at voltages more depolarized than -140 mV resulted in a progressive decline in current amplitude, presumably due to accumulation of inactivation. However, we did develop experimental conditions that enabled a satisfactory evaluation of HEK-Nav1.9 pharmacology by stepping to a voltage that resulted in approximately 20–25% inactivation. Using these conditions, we have been able to show that human and rodent Nav1.9 are inhibited by established sodium channel inhibitors like tetracaine and TC-N 1752, but with considerably lower potency than for other Nav channel subtypes [22, 23, 36]. For example, tetracaine is ~100 fold less potent an inhibitor of Nav1.9 than for Nav1.7 and Nav1.8. It seems unlikely that the difference in potency can be accounted for by differences in the magnitude of inactivation evaluated across Nav channel subtypes (~25% inactivated in Nav1.9 vs ~50% inactivated in other Navs) since we found IC_50_s shifted by no more than 3 fold going from resting to half-inactivated channels (Fig 2B). Contrary to what is known for other Nav channel subtypes, this suggests that the presence of the inactivated conformation is less important for inhibition by local anesthetic agents in Nav1.9.

Despite the lower potency for inhibition of Nav1.9, tetracaine still appears to interact with the canonical local anesthetic binding site, since its potency was reduced by ~6 fold in HEK cells stably expressing the Domain 4 S6 F1592A/Y1599A mutation. We have demonstrated that potency for Nav1.9 inhibition by other local anesthetic-like sodium channel blockers like benzocaine, lidocaine, and mexiletine are also reduced by this mutation. Although TC-N 1752 is a known sodium channel inhibitor [28, 36] it is structurally quite distinct from local anesthetic like agents and its site of interaction has not yet been defined. Our finding in the current study that the inhibitory activity of TC-N 1752 was essentially abolished by the F1592A/Y1599A mutation strongly suggests an interaction of this agent with the canonical local anesthetic binding site.

Our finding that tetracaine, TC-N-1752 and other agents’ exhibit considerably lower potencies for inhibition of Nav1.9 than for other Nav channel subtypes raises the question of whether there are unique residues in the Nav1.9 channel pore that may contribute to this observation. One Nav1.9 specific residue that stood out as a candidate was the charged lysine K799 on the homologous domain 2 S6 helix, which is an asparagine in the equivalent position in all other Nav channel subtypes. This residue is predicted to be spatially opposite to the F1592 residue that contributes to canonical local anesthetic binding site. It is possible that the positively charged lysine limits access or appropriate orientation for interaction for local anesthetic agents like tetracaine, which are predominantly positively charged at physiological pH. Our finding that neutralization of the K799 via replacement with an asparagine resulted in increased potency of tetracaine is supportive of this hypothesis, as is our observation that the potency of the neutral local anesthetic benzocaine is unaffected by mutation of the lysine. Furthermore, when a lysine was inserted into the equivalent pore location of Nav1.7, tetracaine potency was decreased, while benzocaine remained unchanged. While the above explanation is internally consistent for local anesthetic agents and may explain the lower potency of these agents for inhibition of Nav1.9, it does not satisfactorily explain our observation where replacement of the Nav1.9 K799 with asparagine results in an ~6 fold decrease in potency for TC-N 1752 and the reverse equivalent mutation in Nav1.7 increases potency. This molecule is neutral at physiological pH, so a charge based interaction seems unlikely. TC-N 1752 is considerably larger than either tetracaine or benzocaine, and given the finding that mutation of residues on D2S6 and D4S6 reduce potency of TC-N 1752, it is possible that the molecule spans the pore to interact with both of these opposing regions.

In conclusion, the current study has demonstrated that it is possible to stably express functional human and rodent Nav1.9 channels in the widely used HEK-293 heterologous expression system. Our studies have shown that functional Nav1.9 currents expressed in HEK cells exhibit similar properties to that reported for endogenous sensory neuron Nav1.9, but also highlight a variety of biophysical and pharmacological characteristics unique to this channel subtype compared to the other sensory Nav channels. The availability of stable recombinant human and species orthologue HEK-293 cell lines opens up opportunities for more detailed analysis of this historically understudied channel, but also provides a platform on which to build drug discovery programs focused on the identification of novel Nav1.9 modulators for the treatment of pain and gastrointestinal disorders, and potentially others where dysfunctional sensory neurons play a significant role.

## Materials and Methods

### cDNA constructs

All the cDNAs were cloned in either pcDNA3.1 (Invitrogen) or pLNCX2 (BD Bioscience Clontech) mammalian expression vectors using standard molecular biology methods. The cDNA sequences were based on the following Ref Seq#: Human SCN11A (NM_014139), rat *scn11a* (NM_019265) and mouse *scn11a* (NM_11887); human SCNB1 (NM_001037), mouse *scnb1* (NM_011322) and rat *scnb1* (NM_001271045); human SCNB2 (NM_004588), mouse *scnb2* (NM_001014761) and rat *scnb2* (NM_012877).

### Cell line generation

Human β1 and β2 cDNA which were linked with 2A linker were stably expressed in HEK-293 cells. The expression level of β1 and β2 was assessed by qPCR to select a cell line which overexpressed both β1 and β2. This cell line named as HEK-293-βs was used as the base cell line for rest of the human cell line generation. Human Nav1.9, Nav1.9 F1592A/Y1599A, Nav1.9 K799N, Nav1.7 and Nav1.7 N945K channel cDNAs were stably expressed in the HEK-293-β1/β2 cell line, and mouse and rat Nav1.9 cell line were stably expressed in HEK-293-mouse β1/β2 and HEK-293-rat β1/β2. Methods of stable cell line generation were as described in McCormack et al., 2013 [37]

### Electrophysiology

Whole-cell voltage clamp recordings were performed using PatchXpress automated patch clamp eletrophysiological platform (Molecular Devices, LLC) at room temperature (22–24°C). Extracellular solution contained (in mM): NaCl 135, CaCl_2_ 2, KCl 5.4, MgCl_2_ 1, Glucose 5, Hepes 10 (pH 7.4 with NaOH); and intracellular solution (in mM): CsF 135, CsCl 10, NaCl 5, EGTA 5, and Hepes 10 (pH 7.4 with CsOH). 300 nM tetrodotoxin was included in the extracellular solution to block endogenous fast inward Nav currents when recording Nav1.9 or Nav1.9 mutant cell lines, and 500 μM GTP-γ-S is in the intracellular solution to increase the current density for Nav1.9. Nav channel-expressing cells were grown to ~ 50 to 80% confluence and harvested by trypsinization. Trypsinized cells were resuspended in growth media at 2 x 10^6^/5mL and allowed to recover at room temperature for 1 hour before use. Cells were spun down and resuspended in extracellular solution at a concentration of 1 x 10^6^ cells/mL. To determine the voltage dependence of activation, cells were clamped at membrane potential of -140 mV and currents were measured during a 40 ms depolarizing test pulse to potentials from -140 mV to +40 mV in 10 mV increments. To determine the voltage dependence of steady-state inactivation, cells were clamped at a membrane potential of -140 mV followed by a 100 or 500 ms conditioning prepulse to potentials from -140 mV to + 40 mV in 10 mV increments and then a 40 ms test pulse to -40 mV. For pharmacology studies all compounds were dissolved in DMSO to make 10 mM-1M stock solutions, which were then diluted into extracellular solution to obtain the final concentrations desired. The final concentration of DMSO did not exceed 0.3%, which was found to have no significant effect on sodium currents. Test compound effect were evaluated using a protocol in which each cell was clamped at -120 mV that normally inactivated ~25% of available channels and sodium currents elicited by 40ms pulses to -40 mV were sampled every 20 s for 3–5 mins at a frequency of 0.05 Hz.

### Data Analysis

Data was acquired and analyzed using DataXpress 2.0 (Molecular Devices, LLC) and GraphPad Prism (GraphPad Software, Inc.). Sodium currents from activation were converted to sodium conductance [G = I/(V-V_rev_)] and plotted as a function of test potential using the Boltzmann equation [G/Gmax = 1/ (1 + exp((V_0.5_-V)/k)] to give values for V_0.5_ (potential causing half-maximal activation) and k (slope factor). Similarly, currents from steady-state inactivation were also plotted as a function of prepulse potential and fitted to the Boltzmann equation. Concentration response curves were generated from at least 4 different test concentrations (n ≥ 3) using logistic equation [% inhibition = 100/(1+10^((LogIC_50_-X)*HillSlope)))] to calculate IC_50_s for each compound. Statistical analysis was calculated by Student’s *t* test, and P< 0.05 indicates a significant difference. All data are presented as mean ± SEM

## Supporting Information

S1 Fig Comparison of time course of recovery from inactivation for Nav1.9 and Nav1.9 K799N, induced by either a 100 ms (circle) or 5 s (triangle) depolarizing voltage step to 0 mV. Recovery was assessed by applying a 40 ms test pulse to -40 mV after variable periods at -140 mV. Data was fit with a two phase exponential equation with fitted fast and slow time constants shown in Tables 3 and 4.
(TIF)
