# Supplementary figures and images for: Biophysical and Pharmacological Characterization of Nav1.9 Voltage Dependent Sodium Channels Stably Expressed in HEK-293 Cells
Source: PLoS One. 2016 Aug 24;11(8):e0161450. doi: 10.1371/journal.pone.0161450 (PMC4996523; doi:10.1371/journal.pone.0161450)

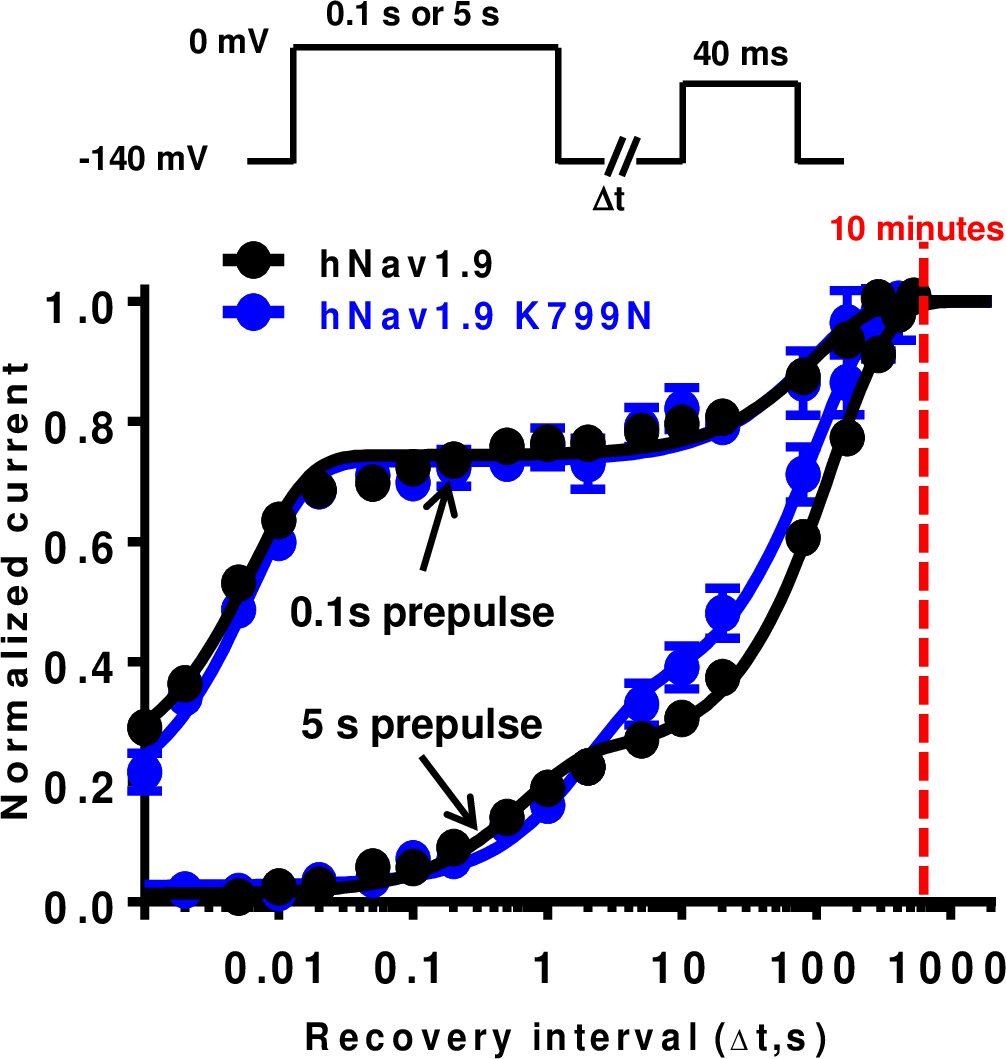

Supplement: S1 Fig — Recovery was assessed by applying a 40 ms test pulse to -40 mV after variable periods at -140 mV. Data was fit with a two phase exponential equation with fitted fast and slow time constants shown in Tables 3 and 4. (TIF) [file pone.0161450.s001.tif]
